# A high-resolution bovine mitochondrial co-expression network

**DOI:** 10.1242/bio.061630

**Published:** 2025-02-03

**Authors:** Pâmela A. Alexandre, Kate Keogh, Antonio Reverter, Nicholas J. Hudson

**Affiliations:** ^1^ Agriculture and Food Department, Commonwealth Scientific and Industrial Research Organisation, Brisbane, Queensland 4067, Australia; ^2^ Animal and Bioscience Research Department, Teagasc Grange, Dunsany, Co. Meath C15 PW93, Ireland; ^3^ School of Agriculture and Food Sustainability, The University of Queensland, Queensland, Gatton QLD4341, Australia

**Keywords:** Beef cattle, Partial correlation, RNAseq, Organelle, Feed efficiency

## Abstract

The mitochondrion is a sophisticated, versatile, and dynamic organelle whose function is incompletely understood. Intending to provide a framework for mitochondrial visualisation and interpretation of genome-wide molecular data, we reverse-engineered a co-expression network whose final structure represented mRNA encoding more than half of the entire mitochondrial proteome. We drew upon 723 RNA-seq data sets representing 91 tissues and cell types from 441 individual cattle. A mitochondrial landscape was formed comprising a main network and many smaller sub-networks. One of the discrete sub-networks contains all 13 mRNA (e.g. *MT-ND1*, *MT -CYTB*, *MT -COX2*, *MT -ATP8*) plus 15/22 tRNA (e.g. *MT-TT*) encoded by the mt-genome itself, indicating some independent regulation from the nuclear genome with whom it must cooperate. Intriguingly, this mtDNA sub-network also contains a single nuclear-encoded gene, that of *PDHA1. PDHA1* encodes a subunit of the pyruvate dehydrogenase complex that governs the conversion of pyruvate to Acetyl CoA. This enzyme is extremely influential, representing the fundamental cellular connection between the ancient, conserved pathway of glycolysis that occurs exclusively in the cytoplasm, and the TCA cycle that occurs within the mitochondrial matrix. To demonstrate the downstream utility of our approach, we overlaid *Longissimus dorsi* muscle transcriptome data from differentially feed efficient Charolais and Holstein Friesian cattle. This approach highlighted expression patterns sensitive to both breed and diet in a complex manner. An analytic advantage of this approach is that relatively subtle (<2-fold) but coordinated changes that may be overlooked by conventional gene-by-gene significance testing become readily apparent. Finally, intending to understand the transcriptional regulation of mitochondrial function more thoroughly, we engineered a network built with transcription factors in addition to those mRNA encoding mitochondrial proteins. Here, a set of influential nuclear hormone receptors (e.g. *PPARA*) are enriched among the most highly and/or well-connected TF.

## Teaser

**Summary:** Characterising the architecture of the mitochondrion using highly conserved patterns of mRNA co-expression.

## INTRODUCTION

Cellular metabolism underpins the physiology of whole organisms and their component tissues. In a healthy organism, an array of metabolic pathways governing the major energy transactions are regulated by an intricate system of molecular checks and balances. The bioenergetic aim is to ensure cellular energy availability (via ATP synthesis) can meet the demands of cellular energy expenditure (via ATP hydrolysis). This regulation, which involves informational crosstalk across multiple levels of biological organisation, is a fundamental component of bioenergetic homeostasis. When the regulation of these foundational cellular pathways goes awry (through mutation, ageing, exposure to toxins, forced anaerobiosis and so on), disease or even death may follow.

The mitochondrion is the sub-cellular seat of aerobic metabolism in the Eukaryotes. This highly conserved organelle is responsible for harvesting the chemical energy derived from food (or endogenous stores) and combusting it in the presence of oxygen to produce ATP. In turn, ATP hydrolysis ‘foots the bill’ for all energy-demanding processes in cells, such as muscle contraction, maintenance of trans-membrane ion gradients and a host of biosynthetic reactions. The mitochondrion also has roles beyond bioenergetics, including cell cycle control and the regulation of programmed cell death. Across the Tree of Life, the mitochondrion is strategically modified in various ways (particularly via cell and tissue content but also through some bespoke modifications in function) to adapt to various biological challenges. These challenges are set by the demands of highly specialised tissues, the physiology of individuals with different capabilities and the ecological niches inhabited by different species.

The avian world is illustrative here, where highly athletic hummingbirds and highly feed-efficient but sedentary broiler chickens occupy two ends of a metabolic spectrum; the *pectoralis* (wing flapping) muscle mitochondrial contents of these two species differ by almost 20-fold, being 35% (Mathieu-Costello et al., 1992) and 2% (Soumeh et al., 2024) respectively. An analogous phenomenon can be observed in human sprinters versus marathon runners, where large differences in mitochondrial content derive from a combination of genetics and environment (i.e. training) (Essen-Gustavsson and Henriksson, 1984; Holloszy, 1975; Secher et al., 1984). On an individual basis, one can also contrast tissues with very different roles. Cardiac muscle, for example, must meet the aerobically met demand for sustainable muscle contraction whereas white adipose tissue's primary role is energy storage, not combustion. In mammals, the heart has a total mitochondrial content of about 25% (Barth et al., 1992) whereas white fat has a cytoplasmic mitochondrial content of 14% (Loncar et al., 1988). The overall value for fat tissue is far lower (probably <1%) as the cytoplasmic portion represents only a tiny fraction of an entire adipocyte. Function on a per unit mitochondrial mass basis can also be modified – for example, thermogenic brown adipose tissue replaces the mitochondrial ATP synthase enzyme with an alternate pore-forming protein UCP1. This pore serves to promote the futile cycling of protons in and out of the matrix without connecting the flow to a molecular rotor, thereby liberating heat in place of ATP.

The molecular regulation of both mitochondrial content and function to enact these various adaptations involves altering patterns of transcription for a large set of genes (∼1500) distributed across two structurally independent genomes, the vast majority of which reside in the nuclear genome. The maternally inherited mitochondrial genome encodes only 37 genes – 13 protein-coding, 22 tRNA and two rRNA. Clearly, these two genomes must cooperate to produce the final functioning organelle. Our knowledge of how this occurs is incomplete, although examples of both antero- (nuclear to mitochondrial) and retrograde (mitochondrial to nuclear) communication have been characterised (Garesse and Vallejo, 2001; Ryan and Hoogenraad, 2007). Key molecules underpinning mitochondrial biogenesis include regulators of mtDNA transcription (such as *TFAM* and *TFB2M*), regulators of nuclear-encoded mitochondrial proteins (e.g. *NRF1* and *NRF2*) and coordination between the two (e.g. *PPARGC1A*).

Here, we used a co-expression network approach aimed at characterising the core structural and functional relationships in mitochondria, using a vast multi-tissue RNAseq resource in cattle as our exemplar mammalian system (Fang et al., 2020). The PCIT network inference algorithm of (Reverter and Chan, 2008) was deployed, which computes partial correlations for all mRNA trios in the system. PCIT is a ‘bottom-up’ data-driven method that does not force a scale-free topology onto the network; this feature will only emerge if it really exists. Our analysis here differs from previous co-expression network reconstruction in mammalian tissues e.g. (Hudson et al., 2009) through an explicit focus anchored on the mitochondria. This has allowed us to generate a comprehensive high-resolution picture of this organelle in unprecedented molecular detail. Its overall topology creates biological insights not possible through other (non-graph theory) approaches, such as identifying clusters of likely co-regulated or co-located encoded proteins and making new functional predictions for some poorly annotated transcripts.

Deploying a co-expression approach across many tissues and biological circumstances will leave intact only those pairwise connections that are highly conserved. Associations that exist in only a subset of experimental states will by definition be lost. This is a deliberate strategy as we were motivated to produce a resource whose final structure would apply as widely as possible. Nevertheless, this does not mean the network cannot shed light on particular biological circumstances. For example, changes in activity of targeted biochemical processes can be detected by differential expression (DE) of some of the resultant mRNA clusters, whose collective function can be predicted through the use of enrichment statistics. To demonstrate our networks' potential, we overlay DE fold change values from a cattle feed efficiency trial and explore the new insights. Our mitochondrial networks are freely available as Systems Biology community resources. These .cys files can be easily visualised, navigated and further mapped within Cytoscape freeware to enable metabolic interpretation of transcriptomic and proteomic data sets from any Eukaryotic species.

## RESULTS AND DISCUSSION

### Co-expression network of the mRNA encoding the mitochondrial proteome

**Fig. 1.**
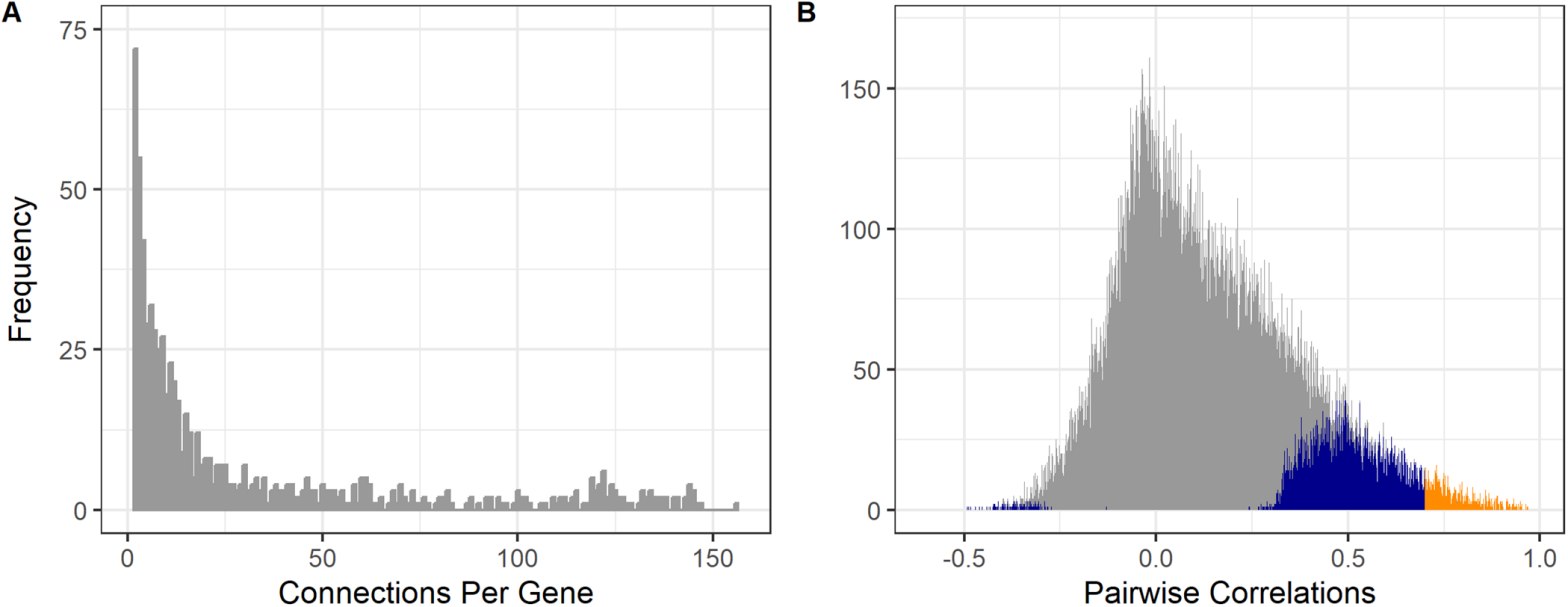
Frequency distribution of (A) the number of connections per gene (degree) in the mitochondria co-expression network comprising 872 nodes and 12,445 edges, and (B) all pairwise correlations among all tested genes with significant correlations stronger than ±0.7 represented in orange and the remaining significant correlations represented in blue.

A robust high-resolution mitochondrial co-expression network comprising 872 nodes, 12,445 edges and an emergent scale-free topology has been reverse-engineered using the PCIT algorithm (Reverter and Chan, 2008) based on 723 RNA-seq data sets representing 91 tissues and cell types from 441 individual taurine cattle (Fang et al., 2020). A scale-free network is characterized by a heavy-tailed degree distribution in which a few genes present a high number of connections to other genes (hubs) and many genes present a small number of connections to other genes (Fig. 1A). As each node denotes an mRNA, a little over half of the ∼1500 transcripts thought to encode the entire mammalian mitoproteome are represented (Rath et al., 2021), along with a set of relationships among them deemed significant by the concept of partial correlation (Fig. 1B). In the current data set, subsequently filtering only those significant correlations greater than±0.7 served to screen out all the negative correlations in the system. An exclusive set of positive correlations is perhaps consistent with the coordinated upregulation in transcriptional activity required for enhanced ATP production, for example.

**Fig. 2.**
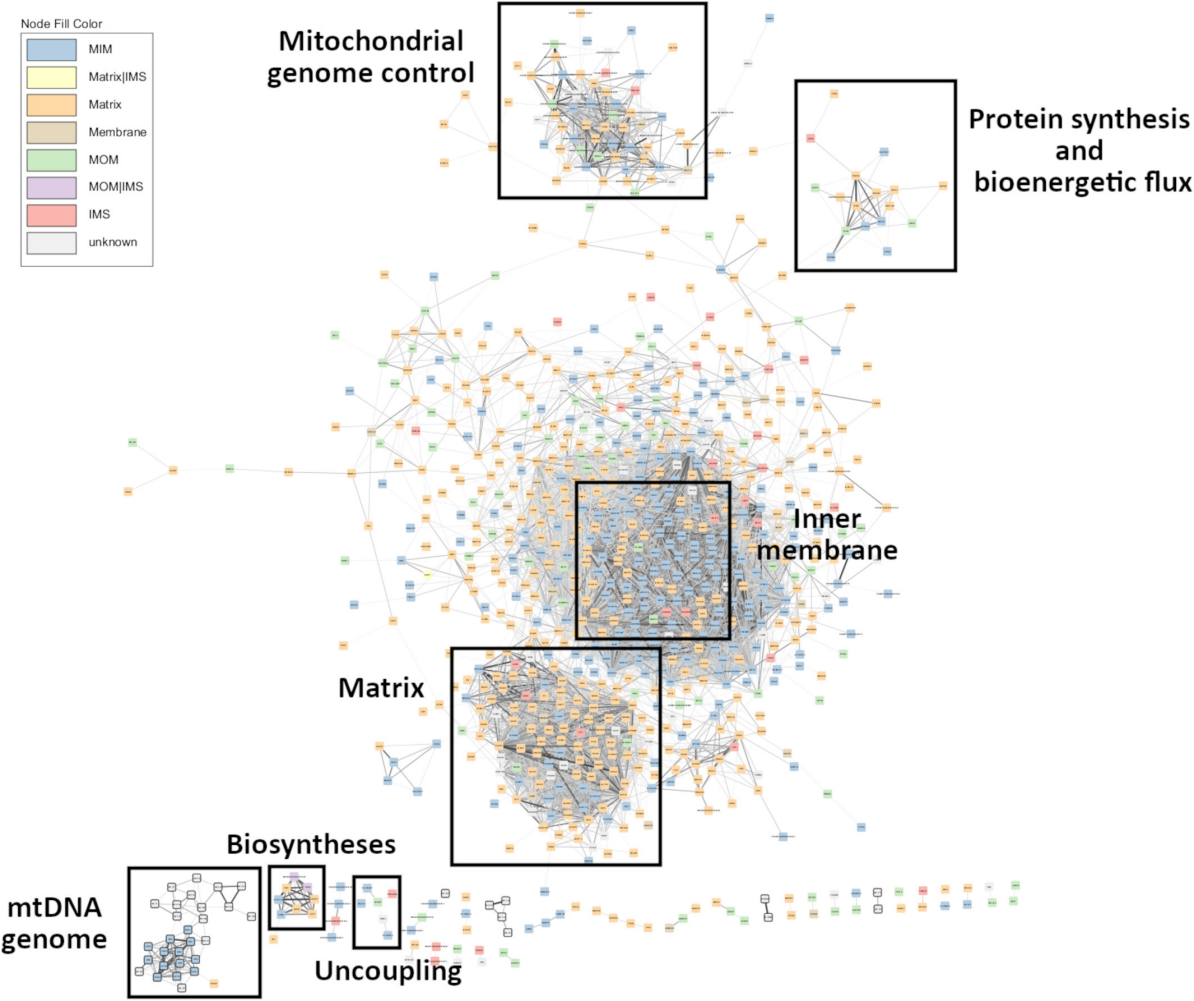
**Mitochondrial co-expression network.** Squared nodes represent 872 mRNA coloured based on cell localization according to MitoCarta 3.0, namely matrix, inner membrane (MIM), intermembrane space (IMS), outer membrane (MOM), mitochondrial membrane, or unknown. Mitochondrial encoded genes are highlighted by black borders. Edges thickness represents the strength of the correlations. Modules of genes related to similar biological processes are highlighted. The largest modules are dominated by mRNA encoding matrix and inner membrane proteins, respectively. Sub-networks include those comprising mRNA encoded by the mtDNA genome; biosynthetic reactions; uncoupling; protein synthesis and bioenergetic flux; and mitochondrial genomic control.

While relatively small mitochondrial modules have been previously published, either as part of a focussed attempt (Chen et al., 2014) or alternately within the broader context of attempting to capture the co-expression arrangements among many cellular compartments and biochemical processes e.g. (Hudson et al., 2009), the network described here is, to the best of our knowledge, the first of its kind in providing a very comprehensive mitochondrially anchored perspective. The network's pairwise connections represent highly conserved gene–gene interactions across the tissues included in this analysis and the biological circumstances of the different animals represented, resulting in a general mitochondrial model on which data from different experiments can be overlayed to generate insights. Among other things, the topology of the network allows the identification of highly interconnected clusters or ‘modules’ (Fig. 2). These modules represent biological processes comprising sets of genes that are functionally related in line with the ‘guilt by association’ heuristic (Wolfe et al., 2005) and part of a tightly co-regulated process, pathway or structure.

One can therefore make *de novo* predictions regarding the biology of unannotated transcripts or encoded proteins that happen to fall within a particular cluster. In general, we found that those mRNA encoding proteins localised to the matrix (such as *ACO2* encoding the enzyme that catalyses the second step of the TCA cycle) are generally found in a separate part of the network than those localised to the inner membrane (such as various components of the electron transport chain, namely *NDUFA/B/C* family members, ATP synthase family members and *UQCR* family members, Fig. 2).

At this point it is important to remember that ATP synthesis is achieved in the mitochondrion by splitting hydrogen atoms into their component electron (e^−^) and proton (H^+^) and sending these subatomic particles on temporary, independent journeys. A series of specialised mitochondrial protein complexes called the electron transport chain drive this process. The electron is passed along the chain while the proton is pumped through the chain past the membrane and into the inner membrane space. Protons possess a charge and are also the basis of pH, so this unidirectional proton pumping creates a powerful ‘dual origin’ electrochemical gradient. The gradient is dissipated in a controlled manner via the ATP synthase enzyme complex that acts as a pore – in effect coupling the passage of protons back into the matrix to the rotation of a specialised molecular motor.

ATP Synthase Peripheral Stalk Subunit D (*ATP5PD*) presents the second higher number of connections within our network (156 connections). It is a component of the mitochondrial ATP synthase that comprises two interconnected complexes: the soluble catalytic core (*F*_1_) and the membrane-spanning component (F_o_), including a proton channel. The *F*_1_ complex contains five subunits (alpha, beta, gamma, delta, and epsilon), while the F_o_ complex, housing the proton channel, likely consists of nine subunits (a, b, c, d, e, f, g, F6, and 8). In our network, we can find directly connected to *ATP5PD* the following mRNAs likely involved in encoding various subunits of the *F*_1_ and *F*_o_ complexes: *ATP5F1A, ATP5PB, ATP5F1B, ATP5MC3, ATP5MJ, ATP5ME, ATP5F1C, ATP5F1E, ATP5PO, TP5MK, ATP5PD, ATP5PF* and *ATP5IF1*.

The H^+^ are initially delivered to the electron transport chain by two donor molecules (NADH and FADH_2_) that in turn are synthesised by the TCA cycle in the mitochondrial matrix. In our network, the most connected transcript (157 connections) together with a mitochondrial ribosome protein (*MRPL37*) is *NDUFC2* (NADH:Ubiquinone Oxidoreductase Subunit C2), an accessory subunit of mitochondrial respiratory chain Complex I (NADH dehydrogenase), is essential for complex assembly, though not directly involved in catalysis. Complex I facilitates the transfer of electrons from NADH to the respiratory chain and is composed of several subunits. Directly connected to *NDUFC2* in our network we have *NDUFS7, NDUFB7, NDUFS2, NDUFA3, NDUFB3, NDUFB10, NDUFB2, NDUFS6, NDUFV1, NDUFC1, NDUFA8, NDUFB5, NDUFA12, NDUFB6, NDUFA13, NDUFB4, NDUFAF3, NDUFA5, NDUFA11, NDUFA6, NDUFA4, NDUFB9, NDUFS8, NDUFS5, NDUFA2, NDUFS3* and *NDUFC2*.

**Fig. 3.**
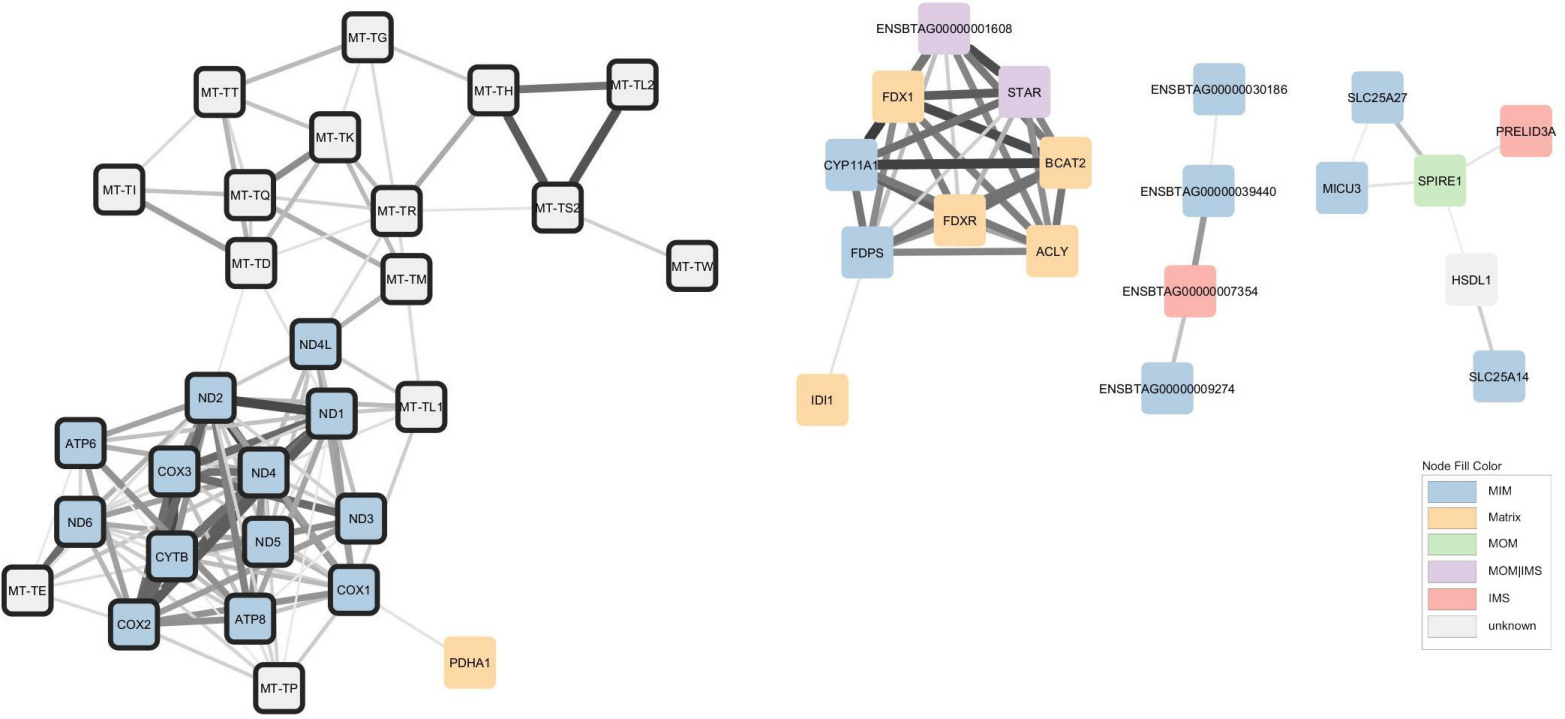
**Mitochondrial co-expression network extract - mtDNA genome, biosynthesis and uncoupling modules.** Nodes are coloured based on cell localization according to MitoCarta 3.0, namely matrix, inner membrane (MIM), intermembrane space (IMS), outer membrane (MOM), or unknown. Mitochondrial encoded genes are highlighted by black borders. Edges thickness represents the strength of the correlations.

Apart from the two largest modules of genes, several of sub-networks were created (Fig. 2). One of the sub-networks contains all 13 mRNA (e.g. *MT-ND1*, *MT -CYTB*, *MT -COX2*, *MT -ATP8*) plus 15/22 tRNA (e.g. *MT-TT*) encoded by the mt-genome itself (Fig. 3). Intriguingly, this mtDNA sub-network also contains a single nuclear-encoded gene, that of *PDHA1. PDHA1* encodes a subunit of the pyruvate dehydrogenase complex that governs the conversion of pyruvate to Acetyl CoA. This link represents the fundamental cellular connection between glycolysis (that occurs exclusively in the cytoplasm) and the TCA cycle (that occurs exclusively in the mitochondrial matrix). The clustering analysis therefore indicates we can detect the long shadow cast by the mitochondrion maintaining some independent control over its vestigial genome, despite the fact that in practice two genomes must cooperate to build the final organelle. Known transcriptional regulators of the mtDNA genome such as *TFB1M, TFB2M, TEFM* and *MTERF2* are not members of the mtDNA sub-network but rather form part of the main network (along with numerous other nuclear encoded mitochondrial proteins).

The two mtDNA-encoded ribosomal RNA (*MT*-*RNR1* and *MT*-*RNR2*) form an exclusive doublet. Equally, *UCP1* (alias *SLC25A27* or thermogenin) is found in a small module with *UCP5* (alias *SLC25A14*). Another noteworthy sub-network comprised mRNA encoding biosynthetic pathways of several major macromolecular classes, namely cholesterol and steroid synthesis (*STAR*, *FDPS, FDX1, FDXR* and *CYP11A1*), *de novo* fatty acid synthesis (*ACLY*) and branched chain amino acid synthesis (*BCAT2*). Also present in this module is an unannotated transcript, ENSBTAG00000001608 whose encoded protein we suggest is likely involved in one of these biosynthetic pathways or similar.

**Fig. 4.**
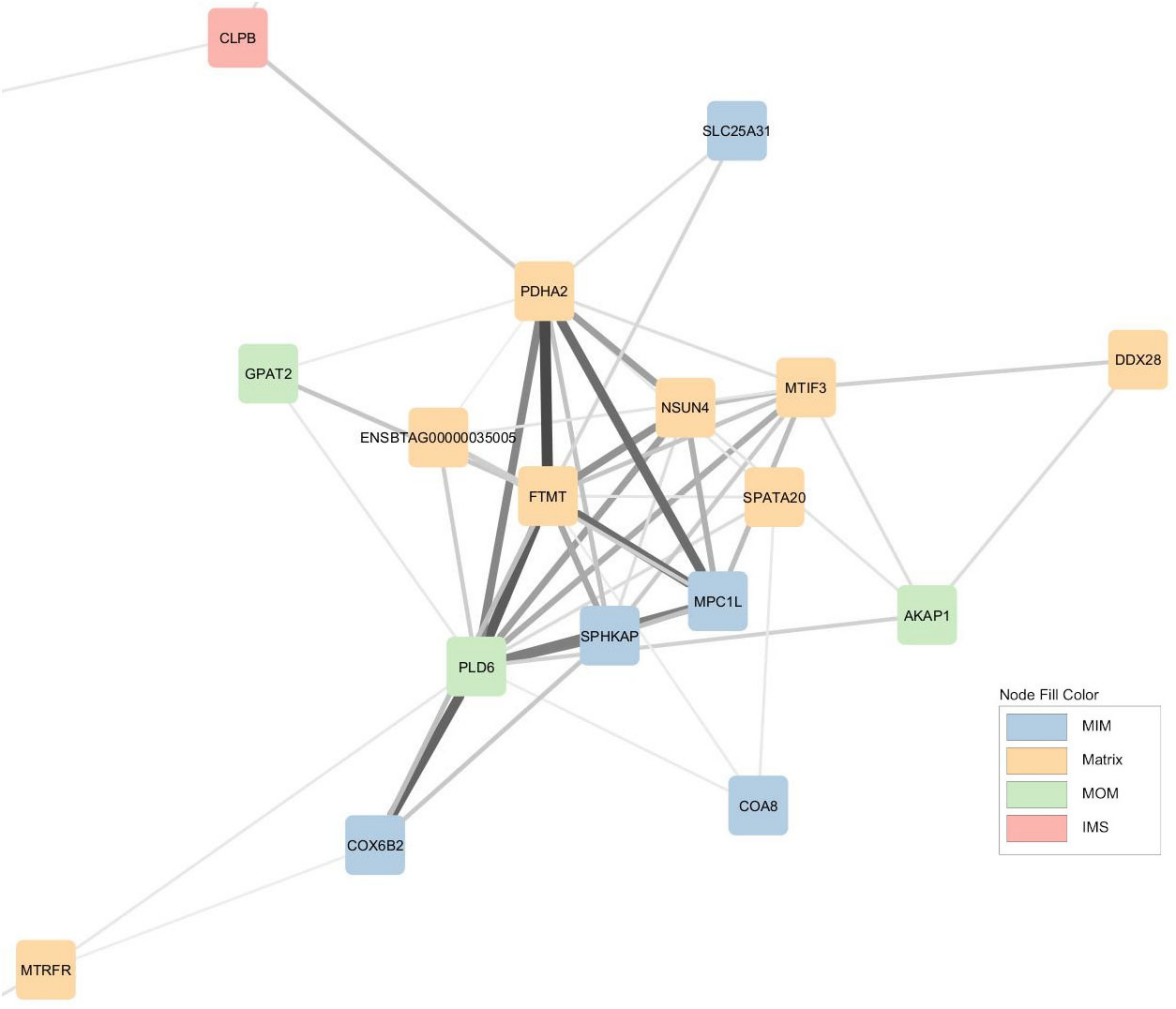
**Mitochondrial co-expression network extract - protein synthesis and bioenergetic module.** Nodes are coloured based on cell localization according to MitoCarta 3.0, namely matrix, inner membrane (MIM), intermembrane space (IMS) or outer membrane (MOM). Edges thickness represents the strength of the correlations.

Another prominent module (Fig. 4) contains mRNA-encoding proteins involved in the regulation of protein synthesis via ribosome assembly (*DDX28*), translation initiation (*MTIF3*) and ribosomal RNA methyltransferase (*NSUN4*). The same module contains mRNA encoding protein involved in bioenergetic flux via pyruvate transport into the mitochondria (*MPC1L*), ADP/ATP transport into and out of the mitochondria (*SLC25A31*) and oxidative phosphorylation (*COX6B2*, *COA8* and *FTMT*).

**Fig. 5.**
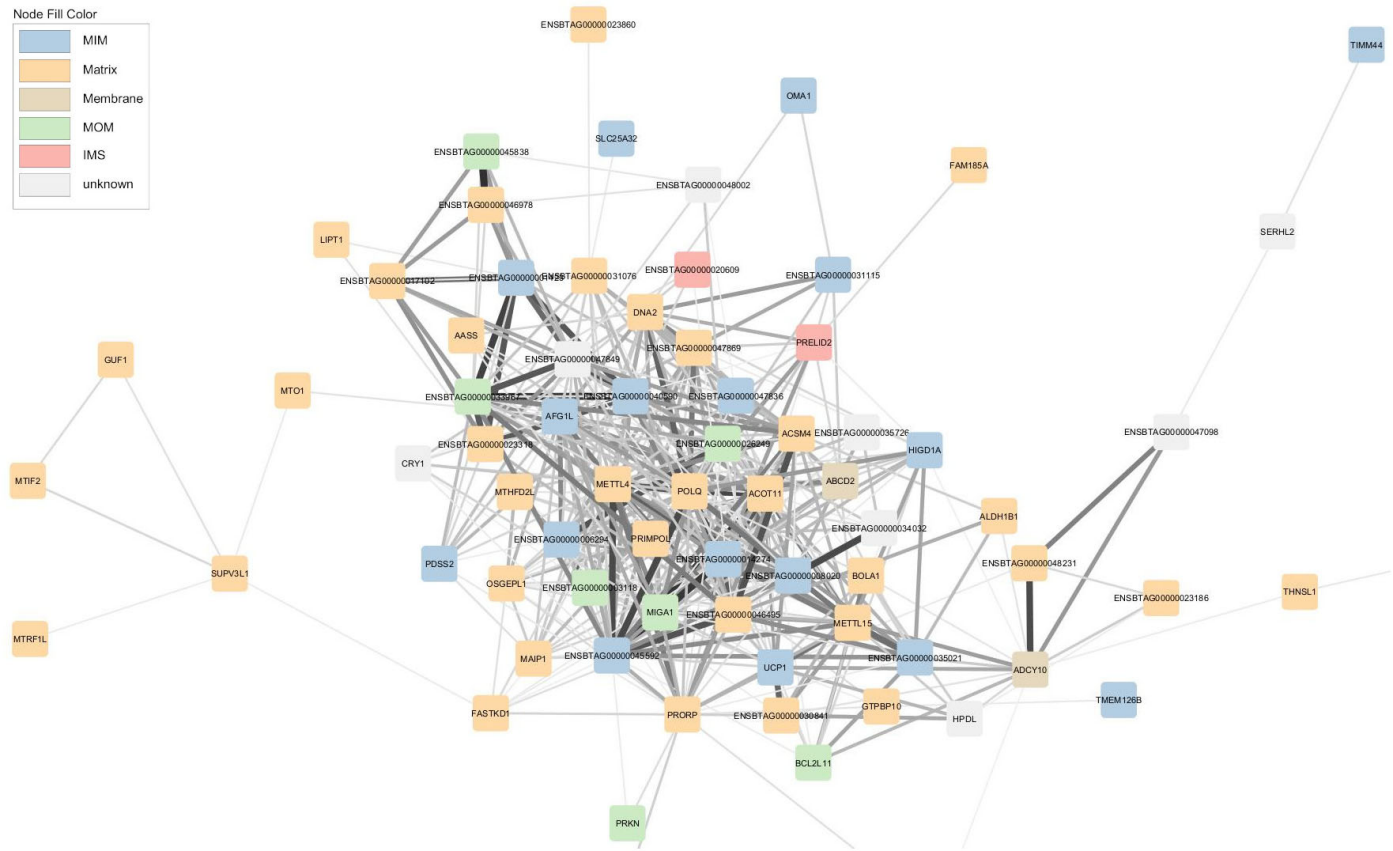
**Mitochondrial co-expression network extract - mitochondrial genome control module.** Nodes are coloured based on cell localization according to MitoCarta 3.0, namely matrix, inner membrane (MIM), intermembrane space (IMS), outer membrane (MOM), mitochondrial membrane or unknown. Edge thickness represents the strength of the correlations.

We also wish to highlight a module containing mRNA encoding proteins involved in the unpacking, synthesis, repair (*PRIMPOL*, *POLQ* and *DNA2*) and replication (*METTL4*, *METTL15*) of DNA, mitochondrial fusion (*MIGA1*), assembly of the electron transport chain (*HIGD1A*, *PDSS2*) and a set of mRNA encoding aspects of FA metabolism (*ACSM4*, *ACOT11* and *ABCD2*). This module (Fig. 5) contains some of the machinery necessary to regulate the mitochondrial genome. Surprisingly, the mRNA encoding enzymes of beta-oxidation (i.e. the combustive pathway for fatty acids) such as carnitine-o-acetyltransferase (*CRAT*), acyl CoA dehydrogenase (e.g. *ACADVL*), enoyl CoA hydratase (e.g. *ECHS1*), 3-hydroxy acyl CoA dehydrogenase (e.g. *HADH*) and 3-ketoacyl CoA thiolase (*ACAA2*) do not form a tight cluster. This indicates the beta-oxidation pathway can either be flexibly manipulated or that pathway-level regulation is enforced post-transcriptionally.

Given mitochondrial structure and function is largely conserved, this network also represents a resource with broad downstream utility. It particularly lends itself to visualising and interpreting genome-wide molecular data derived from tissues sampled from a wide range of mammals, whether production or wildlife. Having said this, the orthologs from other more distant taxonomic groups (including birds, reptiles, amphibians and even beyond) should also translate well given the general architecture of a mitochondrion is considered essentially uniform (Rea et al., 2010).

### Overlay from differentially feed-efficient cattle

As outlined in the introduction, there are numerous biological contexts where an understanding of how genes encoding mitochondrial proteins cooperate might be of relevance. Examples include biomedical science, human sports performance and the physiology of extreme animal performers such as cheetahs and pronghorn antelopes. In animal production science, understanding the mechanistic basis of feed efficiency via a focus on metabolically important tissues such as gut, liver and the skeletal musculature is a notable example.

Mitochondria are often characterised as ‘the engine of the cell’. This being the case, cellular or tissue mitochondrial content reflects overall engine size. In reviewing a role for the mitochondrion in production animal feed efficiency, data on efficiency phenotyped pig muscle (Fu et al., 2017; Kong et al., 2016; Vigors et al., 2019; Vincent et al., 2015), cattle rumen (Kong et al., 2016), rabbit muscle (Huang et al., 2023) and chicken muscle (Iqbal et al., 2004) and heart (Tinsley et al., 2010) is in line with the theoretical perspective that strategic reductions in spare physiological capacity (such as aerobic capacity governed by mitochondrial function) can be exploited to drive gains in animal feed efficiency, *sensu* (Hudson, 2009; Hudson et al., 2017, 2008; Reverter et al., 2017). Also in line with this perspective is the observation that highly feed-efficient, modern broilers possess one of the lowest muscle tissue mitochondrial contents ever documented (Soumeh et al., 2024), and that production species considered as a functional group have significantly lower muscle contents compared to similarly sized non-production species (Hudson, 2009). The central idea is that supporting physiological capacity does not need to be uneconomic and therefore wasteful. After all, a street car with a small engine has a far greater fuel economy at cruising speed than either a Formula 1 or a drag racer.

On the other hand, mitochondrial data in cattle (Alexandre et al., 2019; Fernandez et al., 2020; Kennedy et al., 2021; Keogh et al., 2023; McKenna et al., 2020, 2021) and broilers (Bottje et al., 2002, 2017; Iqbal et al., 2005; Ojano-Dirain et al., 2005) is more equivocal with regard to the physiological capacity theory. Having said this, an overall interpretation across the major farm animals is challenging due to variation in the definition of feed efficiency itself (e.g. RFI versus gain:feed), not to mention substantial variation in breed, age, sex, diet, tissue of origin (e.g. metabolically important or not), tissue preparation (e.g. whole tissue homogenates versus mitochondrial purifications) and phenotyping technology (particularly mRNA levels, which can increase when mtDNA copy numbers decrease (Kong et al., 2016) (Table 1).

**Table 1.** Summary of evidence for the role of mitochondrial function in influencing production animal feed efficiency

FE	Species	Breed	Sex	Tissue (s)	Preparation	Diet	Age	Mitochondrial signal in HFE animals	Reference
Assumed	Comparative review	Not applicable	Undefined	Skeletal musculature	Whole tissue sections	Undefined	Undefined	Production species possess lower tissue mitochondrial contents (planimetric analysis of electron micrographs) than non-production species at common size	(Hudson, 2009)
RFI	Cattle	Charolais and Holstein-Friesian	Males	Skeletal muscle, *longissimus*	Whole tissue homogenates	High concentrate (growing), zero grazed grass (growing) and high concentrate (finishing)	10 m (∼350 kg)	mRNA encoding the mitoproteome DE in a breed and diet dependent manner	(Keogh et al., 2023)
RFI	Cattle	Simmental	Females	Skeletal muscle, *Longissimus*	Whole tissue homogenates	*Ad lib* concentrate and 3 kg silage per day	15 m (∼375 kg)	mRNA encoding *COX1, ND5, ND6, CYTB* and *COX3* upregulated	(McKenna et al., 2021)
RFI	Cattle	Simmental	Males and females	Skeletal muscle, *Longissimus*	Whole tissue homogenates	*Ad lib* concentrate and 3 kg silage per day	15 m (∼375 kg)	Complex I activity showed an RFI×Sex interaction; HFE animals had less complex IV activity, no differences in content detected	(McKenna et al., 2020)
RFI	Cattle	Angus×Hereford	Males	Skeletal muscle	Mitochondrial preparation	Finishing ration	∼378 kg	Complex II activity higher per g of muscle, RCR higher	(Fernandez et al., 2020)
RFI	Cattle	Nellore	Males	Adrenal gland and pituitary	Whole tissue homogenates	Undefined	Undefined	mRNA encoding *mt-CYTB, mt-ND1, mt-ND2, mt-ND4, mt-ND4L, mt-ND5, mt-ND6, and mt-ATP8* mRNA all upregulated in HFE in adrenal gland, *ATP8* downregulated in pituitary of HFE	(Alexandre et al., 2019)
RFI	Cattle	Hereford×Aberdeen Angus	Males	Rumen epithelia	Whole tissue homogenates	Feedlot conditions, finishing diet	Undefined	mtDNA copy number downregulated, mRNA encoding mitochondrial proteins upregulated	(Kong et al., 2016)
RFI	Cattle	Crossbred Angus and Braunvieh	Males and Females	Blood	Isolated lymphocytes	Undefined	Body weights 260-290 kg	Mitochondrial complex I protein upregulated in Angus steers fed 170 days, Braunvieh steers and Braunvieh heifers, but downregulated in the Angus steers fed 160 days	(Ramos and Kerley, 2013)
RFI	Cattle	Holstein	Females	Liver	Whole tissue homogenates	Industry diet	Undefined	mtDNA copy number higher	(Kennedy et al., 2021)
RFI	Pig	Yorkshire	Males (castrated)	Skeletal muscle, *longissimus*	Whole tissue homogenates	Undefined *ad lib*	Undefined	All 25 DE mitochondrial proteins downregulated	(Fu et al., 2017)
RFI	Pig	Large White	Females	Skeletal muscle, *longissimus*	Whole tissue homogenates	Undefined *ad lib*	67 days (27 kg) grown to 115 kg BW	mRNA encoding mitochondrial proteins and mitochondrial proteins both downregulated	(Vincent et al., 2015)
RFI	Pig	(Landrace×Large White)×Meatline	Undefined	Skeletal muscle	Whole tissue homogenates	Standard commercial diets	28 days	mRNA encoding the mitoproteome downregulated	(Vigors et al., 2019)
RFI	Rabbit	Wannan Yellow	Females	Skeletal muscle, *Longissimus*	Whole tissue homogenates	Basal diet for growing rabbits	35 days	mRNA encoding the mitoproteome downregulated	(Huang et al., 2023)
Assumed	Chickens	Ross 308	Undefined	Skeletal musculature, *pectoralis*	Whole tissue sections	Standard industry diet after free ranging		Modern broilers possess one of the lowest tissue mitochondrial contents (2.1% based on planimetric analysis of electron micrographs) of any recorded species	(Soumeh et al., 2024)
Gain:Feed	Chickens	Cobb Vantress pedigree broilers	Males	Skeletal muscle, *pectoralis*	Isolated mitochondrial preparation	Standard industry diet	6-7 weeks	Respiratory chain complexes (I, II, III, IV) had higher enzyme activity whereas protein abundance (core I, CYT C1, CYT B, COXII and ANT1) was lower	(Iqbal et al., 2004)
Gain:Feed	Chickens	Cobb Vantress pedigree broilers	Males	Heart muscle	Whole tissue homogenates	Standard industry diet	6-7 weeks	CII 70S, ISP, CYT B, CYT C1, COXII and ANT1 protein abundance lower but NAD6C higher	(Tinsley et al., 2010)
Gain:Feed	Chickens	Cobb Vantress pedigree broilers	Males	Skeletal muscle, *pectoralis*	Whole tissue homogenates	Standard industry diet	6-7 weeks	mRNA encoding the mitoproteome significantly upregulated, mRNA encoding the mitochondrially localised progesterone receptor predicted as causal by RIF network algorithm	(Bottje et al., 2017)
Gain:Feed	Chickens	Cobb Vantress pedigree broilers	Males	Skeletal muscle, leg and breast	Isolated mitochondrial preparation	Standard industry diet	6-7 weeks	Complexes I and II had higher activity	(Bottje et al., 2002)
Gain:Feed	Chickens	Cobb Vantress pedigree broilers	Males	Duodenum	Isolated mitochondrial preparation	Standard industry diet	6-7 weeks	Complexes I, II, III and V had higher activity whereas IV had lower activity. 6 out of 7 nuclear encoded proteins were lower (70S(FP), core I, core II, Cyt C, ISP, ATPase alpha) and 3/6 mitochondrially encoded proteins (ND4, ND6-C, COXII) were higher	(Ojano-Dirain et al., 2005)
Gain:Feed	Chickens	Cobb Vantress pedigree broilers	Males	Liver	Isolated mitochondrial preparation	Standard industry diet	6-7 weeks	Complexes I, II, III and IV had higher activity. NAD3, subunit VII, COXII and COXIVB proteins were lower whereas subunit 70 and alpha-ATP synthase proteins were higher	(Iqbal et al., 2005)

**Fig. 6.**
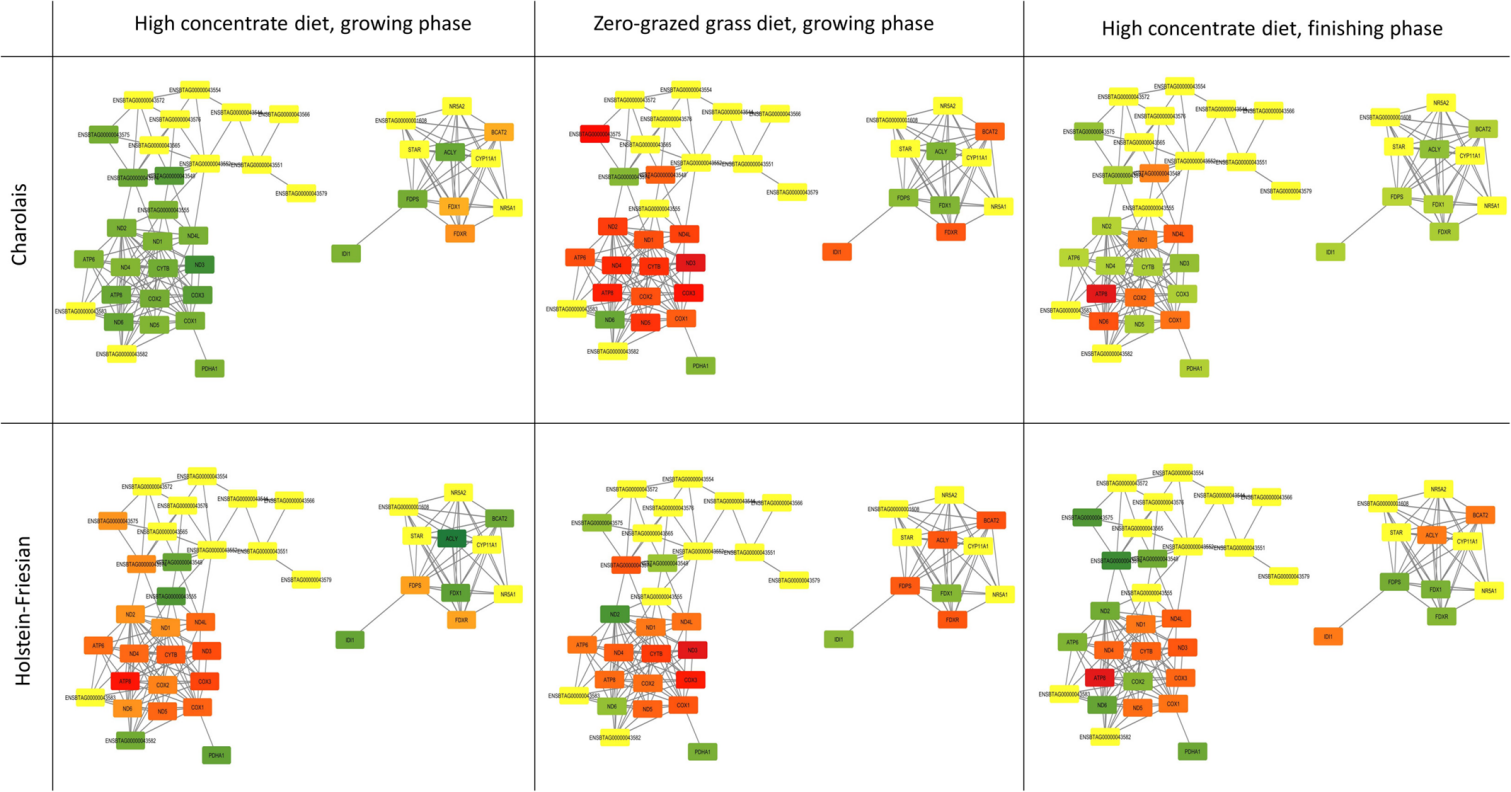
**Overlay of feed efficiency-related gene expression on mitochondrial co-expression network.** Mitochondrial co-expression network node colour continuously mapped to muscle differential expression (log2 fold change) from cattle divergent in feed efficiency across two breeds (Holstein-Friesian and Charolais) and three dietary phases (high concentrate during both growing and finishing phases and zero-grazed grass during the growing phase). Mitochondrial encoded genes as affected by feed efficiency phenotype across breed and dietary contrast. Genes highlighted in green are downregulated in Low-RFI compared to High-RFI, genes in red are upregulated in Low-RFI compared to High-RFI, with genes unaffected by RFI phenotype highlighted in yellow.

Given our particular interest in cattle metabolism, differentially expressed genes in skeletal muscle between cattle divergent for RFI across each dietary phase for each breed (six treatment contrasts) were overlaid onto the mitochondrial co-expression network. Of particular interest were the cluster of mRNA encoded by the mitochondrial genome (module on the left of each panel; Fig. 6). Efficiency based variation in the expression of these mitochondrially encoded genes is dependent on both breed and dietary management. However, *PDHA1*, which encodes a subunit of the pyruvate dehydrogenase complex and is responsible for the conversion of pyruvate to Acetyl CoA was consistently downregulated in the more feed efficient steers in every breed by diet contrast. This is consistent with high cattle feed efficiency being associated with a reduction in flux of Acetyl CoA from glycolysis. A metabolic change like this would be predicted to force the supply of Acetyl CoA to come from the beta-oxidation of energy dense fat instead. Finally, the expression of some mitochondrially encoded genes appear to have no impact on feed efficiency phenotype (yellow nodes in Fig. 6).

An analytic advantage of mapping differential expression (DE) on a co-expression network of this nature is that relatively subtle (<2-fold), but coordinated, changes that might have been overlooked by conventional gene-by-gene significance testing become readily apparent.

### Co-expression network of transcription factors and mRNA encoding the mitochondrial proteome

**Fig. 7.**
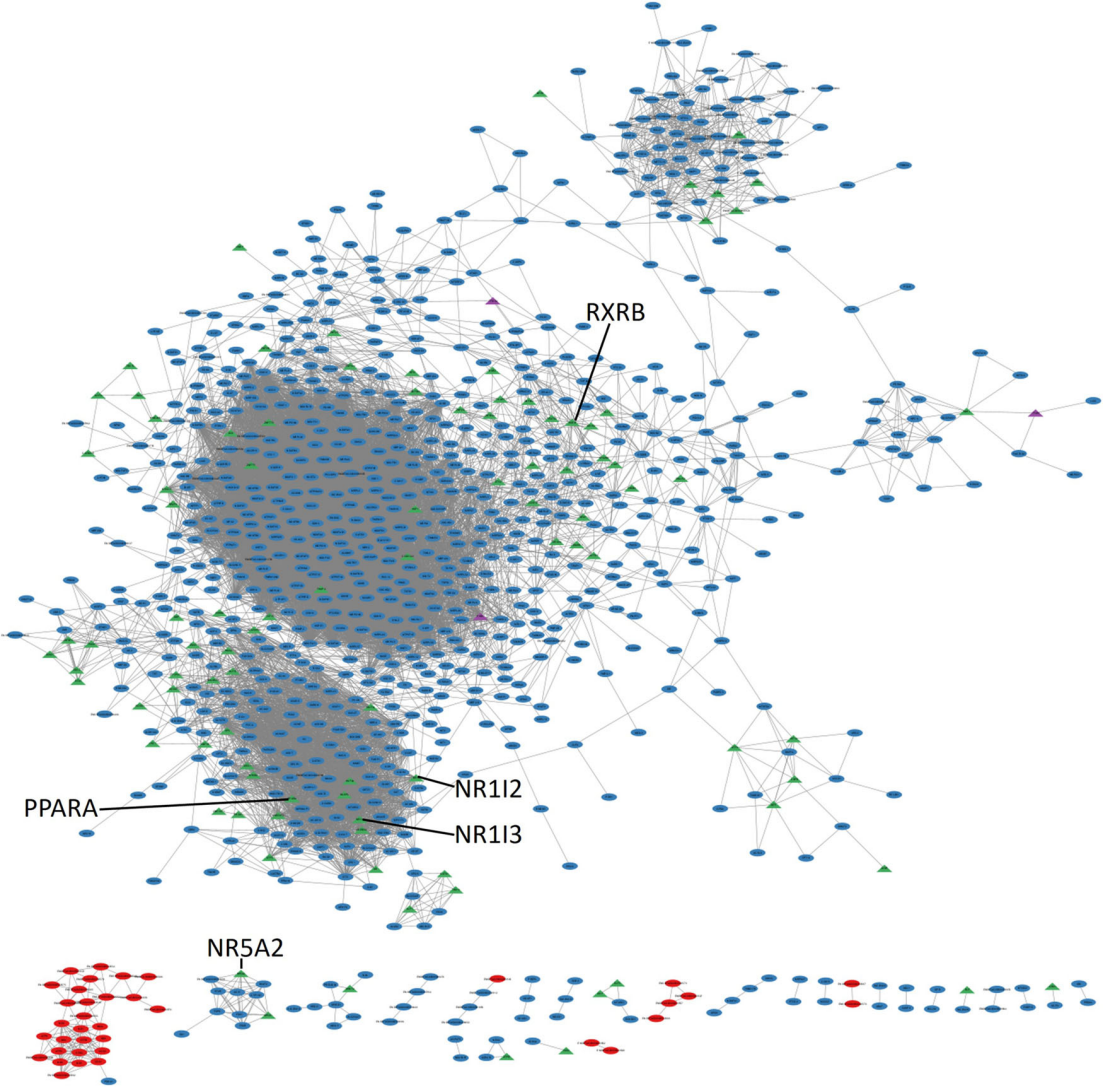
**Mitochondrial and transcription factors co-expression network.** Triangles represent transcription factors (TF) and the remaining genes are represented by circles. Red and blue nodes represented mitochondria or nucleus encoded genes, respectively, purple nodes indicate TF that are also mitochondrial genes and green nodes indicate additional TFs. Influential nuclear hormone receptors are highlighted.

Finally, we attempted to unravel the transcriptional regulation of mitochondrial function more overtly by anchoring the network reconstruction on mRNA encoding TF in addition to those encoding the mitoproteome. The justification is that in gene regulatory networks the major nodes are assumed to be transcription factors (Hobert, 2008). Following clustering and network analysis, a set of influential nuclear hormone receptors (NHR) (e.g. *PPARA*, *NR1I3*, *NR1I2*, *RXRB* and *NR5A2*) are enriched among the most highly and/or well-connected TF across the full landscape (Fig. 7, Tables 2 and 3). Nuclear hormone receptors are a family of mobile, ligand-activated TF that play influential roles in enacting an appropriate gene expression response to metabolic stimuli. *PPARA,* for example, is one of a family of NHR well established as a master regulator of mitochondrial content (Li et al., 2005).

**Table 2.** The top 20 most connected TF in a co-expression network derived from a starting sample of all mRNA encoding TF and all mRNA encoding the mitoproteome

Gene name	Degree	Regulatory function of encoded protein
*THAP4*	108	Nitrate and tyrosine metabolism
***NR1I3***	73	Cytochrome P450 family members
*CCDC124*	70	Cell division
***HNF4A***	63	Cholesterol, glucose and lipid homeostasis
*SCX*	56	Predicted role in mitochondrial solute carrier family regulation
*TCF15*	54	Mesoderm formation
*MLXIPL*	53	Glucose dependent TAG synthesis
*ONECUT2*	53	Role in liver metabolism
*PHB1*	48	Mitochondrial respiration
***NR1I2***	48	Cytochrome P450 family members
*HNF1A*	41	Predicted to bind to the gene mHS that encodes a mitochondrial protein
*HSF1*	35	Cytoprotective proteins such as those relating to heat shock and metabolism
*NKX2-5*	34	
*ASCL1*	34	
*SLC2A4RG*	31	Regulator of SLC4 transporter family
*CREB3L3*	31	Communicates with *PPARA*, fatty acid oxidation
***PPARA***	28	Fatty acid oxidation, target genes include *PDK4*
***RXRB***	27	Mediating retinoic acid, mediating *PPARGC1A*
*ZNF771*	23	
*EMX1*	22	

Nuclear hormone receptors with documented influential roles in mitochondrial metabolism are highlighted in bold.

**Table 3.** The top 20 most influential TF (as estimated by radiality) in a co-expression network derived from a starting sample of all mRNA encoding TF and all mRNA encoding the mitoproteome

Gene name	Radiality	Function
*PHB1*	0.983	Mitochondrial respiration
*CCDC124*	0.983	Cell division
*THAP4*	0.983	Nitrate and tyrosine metabolism
*ZNF771*	0.982	
*HSF1*	0.982	Cytoprotective proteins such as those relating to heat shock and metabolism
*CC2D1A*	0.981	
*ZNF414*	0.981	
*SLC2A4RG*	0.981	Regulator of SLC4 transporter family
*ZNF524*	0.980	
*THAP3*	0.980	Can be localised to the mitochondrion
*ZNF580*	0.980	
*SCX*	0.980	Predicted role in mitochondrial solute carrier family regulation
*TCF15*	0.980	Mesoderm formation
***ESRRA***	0.979	Forms complex with *PPARGC1A*, regulation of mitochondrial content
***RXRB***	0.979	Mediating retinoic acid, mediating *PPARGC1A*
*ZBTB48*	0.979	
***NR2F6***	0.979	Regulated *GRPL2* which exerts a protective effect on mitochondria
*NKX2-5*	0.979	
*ZNF775*	0.979	
*MAF1*	0.978	TCA cycle disruption in mutant mouse strains

Nuclear hormone receptors with documented influential roles in mitochondrial metabolism are in bold.

By way of caveat, it is worth remembering that some key mRNA will not make it onto a co-expression network of this nature. If a given mRNA is not strongly correlated to any other mRNA in the system, it will be eliminated. This could be because it responds so (uniquely) strongly to one or more of the experimental treatments under consideration. A good example is given by *PDK4*, which is transcriptionally activated to inhibit the PDH complex. This action suppresses the supply of Acetyl CoA derived from glycolysis and is sometimes deployed to force the supply of Acetyl CoA to come from the beta-oxidation of fat instead. *PDK4* is activated at the gene expression level by perinatal development (Hudson et al., 2009), starvation (Wu et al., 2000), exercise (Pilegaard et al., 2000) and hibernation (Buck et al., 2002), among numerous other treatments. A final example is given by the canonical rate limiters of the TCA cycle, *CS* and *IDH*. The absence of the mRNA encoding these proteins, and many others like them, should not imply they are unimportant to our understanding of the mitochondrion. Rather, it indicates that they do not fit neatly into a box of co-ordinately regulated pathways or processes, at least at the transcriptional level.

In conclusion, a high-resolution mitochondrial co-expression network representing about half the mRNA encoding the entire mammalian mitoproteome has been reconstructed from 723 cattle tissues using PCIT. This network can be used to make robust predictions of the function of unannotated mRNA and to help visualise and interpret high throughput molecular data from any tissue of any vertebrate species.

## MATERIALS AND METHODS

For this study, we used publicly available gene expression data from the Cattle Gene Atlas (Fang et al., 2020) accessible at https://cattlegeneatlas.roslin.ed.ac.uk/. The fragments per kilobase of transcript per million mapped reads (FPKM) data for 24,616 Ensembl genes (based on UMD3.1) originated from 723 RNAseq libraries comprising 91 tissue and cell types of 441 individual animals. To select genes of interest, we used a list of human genes encoding mitochondrial proteins from MitoCarta 3.0 (Rath et al., 2021) and converted them into bovine orthologues using Ensembl Biomart (Kinsella et al., 2011), which resulted in 1214 genes. We also included all bovine-exclusive mitochondria-encoded genes (24), generating a final list of 1238 genes. From those, 1139 genes were present in our gene expression data and were used for downstream analysis (S1 file).

To generate a mitochondrial co-expression network, we ran the partial correlation and information theory (PCIT, Reverter and Chan, 2008) algorithm using all 1139 genes and 713 RNAseq libraries. PCIT tests all possible three-way combinations of genes and only keeps correlations that are significant and independent of association with another gene in the network. This method has been widely used (289 citations in July 2024) to uncover co-expression and co-occurrence in a variety of scenarios, including multi-tissue transcriptome interactions (Weber et al., 2016), time-series data (Lau et al., 2020), detection of key single nucleotide polymorphism (Mott et al., 2022), exploring the role of long non-coding RNAs (Cracco et al., 2024; Liang et al., 2022) and miRNAs (Manaig et al., 2023; Banerjee et al., 2023), microbial function/modulation (Ramayo-Caldas et al., 2021; Mohamed et al., 2023), etc. To further screen for the most meaningful connections, we retained only significant correlations greater than 0.7 or lower than −0.7 (S2 File). After importing the network into the freely available software Cytoscape v.3.10.0 (Shannon et al., 2003), we visualized nodes (genes) and edges (correlation/connections) using yFiles v.1.1.3 plugin (yFiles Layout Algorithms) organic layout algorithm which is based on the force-directed layout paradigm. The cell localization information from MitoCarta 3.0 was used to classify the genes. The network topology was analysed using the NetworkAnalyzer plugin (Assenov et al., 2008).

To demonstrate one possible use for the mitochondria co-expression networks, feed efficiency gene expression data derived from steers divergent for residual feed intake (RFI) were overlaid onto the mitochondrial co-expression network. This dataset was previously reported by (Keogh et al., 2023). Specifically, steers of two contrasting breeds [Charolais (*n*=90) and Holstein-Friesian (*n*=77)] were evaluated for RFI over consecutive contrasting dietary phases. Contrasting dietary phases consisted of (1) a high-concentrate diet during the growing phase; (2) a zero-grazed grass diet during the growing phase; and (3) a high-concentrate diet during the finishing phase. Diet composition for each dietary phase is included in (Keogh et al., 2023). Following a 14-day dietary adaptation period, individual animal intake and live weight measurements were recorded over the three 70-day feeding phases. Upon completion of each dietary phase, individual RFI values were computed for each steer as previously described in (Higgins et al., 2018) and the 12 steers with the highest and lowest RFI values for each breed were selected for *longissimus dorsi* muscle biopsy collection at the end of each of the three dietary phases (Keogh et al., 2023).

*Longissimus dorsi* muscle tissue samples were collected from selected steers divergent for RFI through punch biopsy between the 12th and 13th ribs. RNA was subsequently isolated from biopsy samples and RNA sequencing was performed as previously described in (Keogh et al., 2023). Resultant sequencing reads were firstly checked for quality (FastQC; Andrews, 2010), followed by the removal of sequencing adapters and any low-quality reads (Cutadapt; Martin, 2011) and were subsequently aligned to the bovine reference genome (ARS-UCD1.2; Rosen et al., 2020) using STAR (Dobin et al., 2013). Differential expression of genes was undertaken using the edgeR package (Robinson et al., 2010). Within edgeR, gene expression reads were estimated as counts per million (CPM) and genes that presented at least 1 CPM in at least half of the samples were retained for differential expression analysis. Differential expression analysis was undertaken for six contrasts of RFI phenotype for the two breeds across the three contrasting dietary phases. The log2 fold change of all genes either up- or downregulated in feed efficient versus feed inefficient steers were applied to the co-expression network.

Finally, to explore which bovine transcription factors (TF) could have important roles in mitochondrial processes, we generated a second co-expression network. This time we added to the 1139 mitochondrial genes, all bovine TF listed in the AnimalTFDB 4.0 (Shen et al., 2023) and repeated the PCIT analysis and filters. To make sure we only kept in the final network TFs that were relevant to this study, we excluded TF with more connections to other TF than to mitochondrial genes.

## Supplementary Material

10.1242/biolopen.061630_sup1 Supplementary information

File S1. Bovine orthologs of human mitochondrial genes.

File S2. Mitochondrial co-expression network in text format containing gene to gene association.
