# Supplementary material for: A high-resolution bovine mitochondrial co-expression network
Source: Biol Open. 2025 Feb 3;14(2):BIO061630. doi: 10.1242/bio.061630 (PMC11832118; doi:10.1242/bio.061630)
Supplement: Supplementary information [file biolopen-14-061630-s1.pdf]

File S1. Bovine orthologs of human mitochondrial genes.

Available for download at
https://journals.biologists.com/bio/article-lookup/doi/10.1242/bio.061630#supplementary-data

File S2. Mitochondrial co-expression network in text format containing gene to gene association.

Available for download at
https://journals.biologists.com/bio/article-lookup/doi/10.1242/bio.061630#supplementary-data
